# Uremic Pericarditis in a Patient With Hyperphosphatemic Familial Tumoral Calcinosis: Case Report

**DOI:** 10.1155/carm/3968524

**Published:** 2025-05-29

**Authors:** Rana A. Nabalawi

**Affiliations:** Department of Nephrology, King Abdulaziz University Faculty of Medicine, Jeddah, Saudi Arabia

**Keywords:** chronic kidney disease, hyperphosphatemic familial tumoral calcinosis, NSAID, pain management, uremic pericarditis

## Abstract

Hyperphosphatemic familial tumoral calcinosis (HFTC) is a rare hereditary disorder characterized by defective phosphate homeostasis, leading to ectopic calcium deposition in soft tissues. This case report describes a 41 year-old Jordanian male with HFTC and stage 5 chronic kidney disease (CKD) secondary to nonsteroidal anti-inflammatory drug (NSAID) abuse, who presented with symptoms suggestive of uremic pericarditis (UP). His medical history included multiple excisions for tumoral calcinosis, epilepsy, and hypertension. Upon presentation, the patient exhibited severe retrosternal pain, dyspnea, and signs of renal failure. Laboratory findings confirmed severe anemia, metabolic acidosis, hyperkalemia, hyperphosphatemia, and hypocalcemia. Imaging revealed mild pericardial effusion and echogenic kidneys. Following a diagnosis of UP, the patient was initiated on daily hemodialysis and received blood transfusions and antibiotic therapy. His condition improved significantly, with complete regression of pleural effusion and stabilization of renal function. This case emphasizes the importance of effective pain management in HFTC to prevent the misuse of analgesics like NSAIDs, which can lead to severe complications such as UP. This report serves as a valuable reminder of the intricate relationship between medication management and the worsening of underlying health conditions in patients with HFTC.

## 1. Introduction

Hyperphosphatemic familial tumoral calcinosis (HFTC) is a rare hereditary disorder typically inherited in an autosomal recessive manner. This condition arises due to a defective phosphate homeostasis regulation, resulting in calcium accumulation in soft tissues. HFTC is caused by a deficiency in, or resistance to, fibroblast growth factor-23 (FGF23), a crucial factor in regulating renal phosphate excretion. Specifically, FGF23 inhibits cellular phosphate reuptake in the proximal renal tubules. When deficient, FGF23 leads to increased renal reabsorption of phosphorus [1, 2]. This results in the elevation or inappropriate production of 1, 25-dihydroxy vitamin D, which promotes gastrointestinal absorption of phosphorus and calcium and increases ectopic tissue deposition [2].

The calcium depositions (tumoral calcinosis) typically affect the soft tissues of periarticular regions such as the hips, elbows, and shoulders. These articulations are more exposed to repetitive trauma or prolonged pressure; therefore, they are prone to inflammation, further activating calcification [2]. Moreover, patients experience painful swellings due to hyperostosis in the areas overlying the diaphyses of the tibiae (the most common site) and, less frequently, the ulna, metacarpal bones, and radius. Hence, pain is the hallmark of HFTC and can be highly debilitating, thus altering the quality of life of patients [2].

Uremic pericarditis (UP) is a particular form of metabolic pericarditis observed essentially in patients with end-stage renal disease (ESRD). Although its incidence has decreased dramatically throughout the years, it is still significant in chronic hemodialysis patients, reaching 5%–20% [3]. The association of UP with tumoral calcinosis is very uncommon and was only described as a secondary presentation in hemodialyzed patients [4]. In this report, we discuss the case of a patient with a known history of HFTC and advanced-stage chronic kidney disease (CKD) secondary to nonsteroid anti-inflammatory drugs (NSAID) abuse for analgesia who presented to our hospital with suggestive signs of UP.

## 2. Case Presentation

A 41-year-old Jordanian male with a known history of HFTC presented to our emergency room for acute retrosternal chest pain, dyspnea, and decreased urine output. He was diagnosed with HFTC at the age of 25 years following surgical excision of multiple calcified masses in his hip. He subsequently underwent more than 28 successive excisions of tumoral calcinosis at various joints, including the hip, shoulder, and elbows. The last surgery was performed on the left hip 75 days before the presentation and was complicated by wound abscess, septic arthritis, and osteomyelitis managed with surgical debridement and collection drainage. His medical history was positive for epilepsy, managed with levetiracetam (500 mg daily) and valproic acid (1000 mg daily). The patient received his HFTC diagnosis at age 25, with kidneys functioning at regular levels. Besides, he had stage 5 CKD and a recently diagnosed hypertension, both of which were attributed to Diclofenac abuse of more than 100 mg per day intramuscular for more than 5 years. In this case, the primary purpose of administering Diclofenac was to address pain symptoms resulting from tumoral calcinosis. The patient demonstrated increasing renal dysfunction while continuing sporadic NSAID consumption throughout multiple years until reaching Stage 5 CKD diagnosis. The persistent use of NSAIDs appeared to speed up the development of ESRD and could have worsened the natural course of tumoral calcinosis. He is allergic to ceftriaxone, mesalamine, and sulfasalazine. He received 2 doses of the Pfizer COVID-19 vaccine. Additionally, he was receiving injections of anakinra in the USA, where he was followed for his primary disease (i.e., HFTC). His full medication history included levofloxacin, amlodipine, hydralazine, sevelamer, rosuvastatin, aspirin, and pantoprazole.

The patient was conscious and alert during the presentation, but his general status was altered. His vital signs were recorded: body temperature: 37°C, BP: 168/99, heart rate: 110 bpm, respiratory rate: 25 bpm, SpO_2_: 88%–90% on room air. He reported severe retrosternal pain that forces him to lean forward, with no cough or hemoptysis. Cardiovascular examination showed a pericardial rub and positive pulsus paradoxus, while lung auscultation revealed bilateral fine basal crepitations. Urine output was 400 mL over the last 24 h, and pitting edema (2+) was present on the inferior limbs. Estimates show that the patient typically produces urine in amounts less than 500 mL/day, so the current 400 mL output could be attributed to volume overload instead of dehydration. Skin and mucous membrane examination demonstrated pallor in addition to scattered ecchymoses. The left hip had a restricted range of motion with eventual pus discharge at the surgical incision site. Neurological examination and the gastrointestinal examination were unremarkable, except for mild epigastric tenderness and a soft abdomen without distension. The patient has already received hemodialysis sessions (3 per week) in another center; however, due to persistent pain and insurance expiry issues, he decided to visit our hospital. The patient received standard hemodialysis treatments at another medical center for 4 months through three sessions per week. The patient underwent his last recorded dialysis session 3 days before the hospital visit. The continuous pain, along with the expiration of insurance benefits, led this patient to seek care at our facility.

Laboratory workup showed severe anemia (Hb: 5.7 g/dL, hematocrit 25.7%), metabolic acidosis with high anion gap (17 mmol/L, blood pH: 7.321), hyperkalemia (5.9 mEq/L), hyperphosphatemia (1.69 mmol/L) and hypocalcemia (1.9 mmol/L), in addition to increased blood urea (30 mg/dL) and creatinine (785 mg/dL) levels, and increased pro-BNP (11,825 pg/mL). Liver function tests showed mild transaminitis (AST 45 IU/L, ALT 50 IU/L). The rest of the laboratory findings were unremarkable (Table 1).

To rule out a cardiac or respiratory emergency, a chest x-ray and an ECG showed mild effusion in the left pleura and sinus tachycardia, respectively. Notably, cardiac ultrasound revealed mild pericardial effusion with an ejection fraction of 40%–45%. Kidney and bladder ultrasound showed small and echogenic kidneys (the right kidney measured 8 × 3.5 cm, and the left kidney measured 6.6 × 2.4 cm) with no stones or hydronephrosis and an empty bladder.

Based on the laboratory and imaging findings, the diagnosis of UP was made, and the patient was again started on hemodialysis daily. The medical team first viewed this intervention as an acute method, but outpatient hemodialysis emerged as the subsequent treatment plan after clinical improvement. The sessions lasted 4 h, while ultrafiltration settings used fluid status as the basis for adjustment. He also received 14 units of packed red blood cells for his severe anemia during the hospitalization period, with monitoring of hemoglobin levels post-transfusion. The measured parathyroid hormone (PTH) level reached 625 pg/mL, which demonstrated secondary hyperparathyroidism. During the fourth day of hospitalization, he abruptly developed hematemesis and melena. Consequently, an upper and lower endoscopy was performed by the gastroenterology staff, who identified multiple gastric erosions and colonic ulcers and managed the active bleeding by sclerotherapy. These Findings were consistent with chronic NSAID use, which likely contributed to mucosal damage. Furthermore, blood cultures showed methicillin-sensitive *Staphylococcus aureus* and ESBL *Escherichia coli*.

Following 3 weeks of daily hemodialysis and administration of an intensive antibacterial regimen that included vancomycin and meropenem, the patient's clinical status has demonstrated a significant amelioration, concomitant with complete regression of the previously diagnosed pleural effusion and resolution of symptoms. The patient is scheduled to return to the United States to continue the administration of anakinra for the required duration. He will be subject to routine evaluations to assess his eligibility for potential renal transplantation at a later time point, cardiac evaluation, and consideration for potential living donor transplant options.

## 3. Consent Statement

The patient was given informed consent, which explicitly explained that the participation was voluntary and the participant could at any time retract her consent to participate.

## 4. Discussion

To our knowledge, this is the first reported case of UP and CKD secondary to NSAID abuse in a patient with HFTC. The patient presented with classic manifestations of acute pericarditis, including severe retrosternal pain that was alleviated by leaning forward, pericardial rub, positive pulsus paradoxus, and mild pericardial effusion on cardiac ultrasound. Additionally, the patient's creatinine and urea blood levels were elevated. Given the patient's renal condition and the absence of alternative etiologies for pericarditis, such as viral infection, autoimmune disorders, or cardiac ischemic disease, a diagnosis of UP was considered the most likely. The patient also had high levels of phosphorus in the blood, a hallmark feature of HFTC that can be further exacerbated by CKD. The patient exhibited low calcium levels, possibly due to reduced renal production of 1, 25-dihydroxyvitamin D.

Our case study underscores the challenges of managing pain in patients with HFTC and highlights the critical importance of pain management as a key treatment objective. Upon presentation, the patient was initially treated with anakinra, a recombinant interleukin-1 (IL-1) receptor antagonist purported to provide an anti-inflammatory action and pain relief for both tumoral calcinosis and hyperostosis lesions, notably in HFTC [5, 6]. However, the patient's history of NSAID use resulting in stage 5 CKD, hypertension, and suspected iatrogenic gastric and colonic ulcerations suggests that he had been experiencing chronic, uncontrolled pain that necessitated specialized pain management consultation to prevent such catastrophic outcomes. The pathogenesis of NSAID-induced nephropathy is believed to primarily stem from the inhibition of prostaglandin synthesis and the consequent reduction in vasodilatory effects, which results in significant decreases in renal blood flow and renal medullary ischemia [7]. In patients with painful conditions such as HFTC, requiring long-term use of NSAID, preventative strategies for avoiding renal injury include the use of the minimal effective dose of NSAID for the shortest possible duration, along with strict monitoring of renal function, fluid retention, and electrolyte abnormalities [8]. Moreover, patient education regarding the potential side effects of NSAID intake is crucial to avoid treatment abuse.

The exact pathophysiology of UP is not fully understood; however, it has been suggested that it may result from the accumulation of toxic metabolites and nitrogenous wastes, in addition to unbalances in hydroelectrolytic and acid-base homeostasis. Further contributors to the pathogenesis of UP have been proposed, including hyperparathyroidism, hyperuricemia, hypocalcemia, and viral infections, particularly with cytomegalovirus, influenza, and Coxsackie viruses. Moreover, it was speculated that inflammatory damage to the pericardium may also result from urea- and toxins-triggered immunological responses (i.e., inflammasomes and IL–1–mediated cascades) [9]. On pathology, UP is primarily fibrinous, but it can be serous and rarely hemorrhagic. In its severe form, the fluid is exudative, with high levels of protein and mononuclear cells [3], suggesting an inflammatory origin. In the present case, no pathology investigations of the pericarditis were carried out, which would have comforted the diagnosis.

The main risk of UP is the life-threatening complication of cardiac tamponade. In UP, pleural effusion has a large volume in one-third of UP patients, imposing urgent diagnosis and adequate management with prolonged hemodialysis sessions for up to 2-3 weeks. When the treatment is well conducted, it is usually sufficient and allows complete regression of the pericardial effusion [10]. In case of treatment failure with repetitive hemodialysis sessions, it is recommended to perform pericardiocentesis within 7–14 days. Severe UP with sizable pericardial effusion exposes to a high risk of cardiac tamponade, where an urgent pericardiocentesis is indicated [11]. After the enduring nature of HFTC and ESRD, patients need to receive prolonged clinical observation to detect and handle UP and other sequelae. Patients who previously had UP need regular medical checks because inadequate dialysis or persisting metabolic abnormalities raise their chance of recurrent UP. Identifying recurrence in patients requires regular echocardiographic examinations, biochemical measurements, and dialysis efficiency checks as part of sustained healthcare assessment. NSAIDs must be strictly avoided in all cases, especially when patients have CKD. The combination of IL-1 inhibitors, such as anakinra, with physical therapy treatment produces effective pain management alongside low-dose opioid medications that should be selected and administered by nephrologists. Patient care should integrate the expertise of nephrology professionals with rheumatology services and pain management specialists to provide complete medical support, which decreases disease challenges and reduces medication side effects. Long-term management requires patients to learn proper analgesic use safety, along with regular assessments for eligibility to receive a renal transplant, as essential points.

## 5. Conclusion

This illustrative case highlights the complex interplay between direct pathophysiological mechanisms and the detrimental effects of mismanagement and prolonged misuse of harmful medications in patients with HFTC. Among these morbidities, UP should be considered in the case of advanced-stage CKD, particularly in hemodialyzed patients who present with evocative symptoms and negative signs of other cardiac emergencies. We highlight the necessity of improving pain management in patients with HFTC to prevent the misuse of analgesic drugs, particularly NSAIDs, and their severe, irreversible complications.

## Figures and Tables

**Table 1:** Laboratory investigation findings.

Leukocytes count (NR: 5000–10,000)	Platelets count (NR: 150,000–450,000)	Blood sodium (NR: 135–145 mEq/L)	PTT (NR: 25–35 s)	PT (NR: 11–13.5 s)	INR (NR: 0.8–1.1)	Blood magnesium (NR: 0.65–1.05 mmol/L)	HBV, HCV, HIV serologies
6700	157,000	130	36	14	1.3	0.66	All negative

Abbreviation: NR, normal range.

## Data Availability

The data that support the findings of this study are available on request from the corresponding author. The data are not publicly available due to privacy or ethical restrictions.
